# The effectiveness of acupuncture for chronic pain with depression: A systematic review protocol

**DOI:** 10.1097/MD.0000000000008800

**Published:** 2017-11-27

**Authors:** Ziyi Yang, Ling Zhao, Xianze Xie, Tao Xu, Yutong Zhang, Xing Wang, Jiarong Du, Ziwen Wang, Mengyuan Zhou, Ying Li, Siyuan Zhou

**Affiliations:** a College of Acupuncture and Moxibustion and Tuina, Chengdu University of Traditional Chinese Medicine; b College of Foreign Language & Cultures, Chengdu University of Technology, Chengdu, Sichuan, China.

**Keywords:** acupuncture, chronic pain, depression, protocol, systematic review

## Abstract

**Background:**

Chronic pain is a major public health problem and 30% to 45% of sufferers experience severe depression. Acupuncture is often used to treat both depression and a range of pain disorders. We aim to conduct a systematic review of randomized controlled trials (RCTs) to evaluate the efficacy of acupuncture for patients experiencing chronic pain with depression.

**Methods:**

To identify relevant RCTs, the following databases will be searched electronically from their inception to July 1, 2017: PubMed, MEDLINE, EMBASE, Cochrane Central Register of Controlled Trials, the Allied and Complementary Medicine Database, the Cumulative Index to Nursing and Allied Health Literature, Chinese medical databases, and others. Manual retrieval will also be conducted. RCTs that evaluated acupuncture as the sole or adjunct treatment for patients with chronic pain and depression will be included. The primary outcomes will be based on a visual analog pain measurement scale and the Hamilton Depression Scale. The secondary outcomes will include scores on a numerical rating scale, verbal rating scale, and the Hospital Anxiety and Depression Scale. The study selection, data extraction, and study quality evaluation will be performed independently by 2 researchers. If the data permit, meta-analysis will be performed using RevMan V5.3 statistical software. If the data are not appropriate for meta-analysis, descriptive analysis or subgroup analysis will be conducted. The methodological quality of the included trials will be assessed using the Cochrane risk-of-bias criteria and the Standards for Reporting Interventions in Controlled Trials of Acupuncture checklist.

**Results:**

This study will provide a high-quality synthesis of current evidence of acupuncture for chronic pain with depression from several scales including visual analog pain measurement scale, the Hamilton Depression Scale, a numerical rating scale, verbal rating scale and the Hospital Anxiety and Depression Scale.

**Conclusion:**

The conclusion of our study will provide updated evidence to judge whether acupuncture is an effective intervention for patients suffered from chronic pain with depression.

## 1 Introduction

### 1.1 Description of the condition

Pain is defined by the International Association for the Study of Pain (IASP) as an unpleasant subjective feeling and emotional experience associated with tissue damage or potential tissue damage, which is a combination of physical, psychological, emotional, cognitive, behavioral, and social factors that interact with each other.^[1,2]^ The IASP defines chronic pain as persistent or intermittent pain for more than 3 months.

Pain and depression are 2 of the most critical public health issues facing health care providers today. Chronic pain is predicted to affect up to 10% to 33.3% of adults worldwide.^[3]^ Data from a European population-based study indicate that 19% of individuals suffered from chronic pain [10-point numerical rating scale (NRS) >5], 66% of these had moderate pain (NRS 5–7), and 34% had severe pain (NRS 8–10).^[4]^ Ohayon and Stingl ^[5]^ found a 2011 chronic pain prevalence of 24.9% in Germany. Similarly, it is estimated that depression affects approximately 350 million people globally and 16.2% of Americans at some point in their lifetime.^[6,7]^ Depression is one of the leading causes of disability worldwide and is a significant contributor to increased medical costs and economic burden.^[6,8]^ Unfortunately, pain and depression frequently coexist and this comorbidity is associated with a greater burden to the individual and society than either condition alone.^[9,10]^ Chronic pain decreases patients’ quality of life, which can affect their mood and lead to depression. The occurrence and development of chronic pain are closely related to psychological factors such as anxiety, depression, mood, and stress.^[11,12]^ Statistics show that 30% to 45% of patients with chronic pain suffer from severe depression.^[13]^ One epidemiological survey found that the incidence of depression in patients with chronic pain was 52% and that 65% of patients with depression have symptoms of pain.^[14]^

Depression and pain share biological pathways and neurotransmitters, which has implications for their concurrent treatment. The pain experience involves activation of multiple brain regions and can lead to mental illness and impairments in quality of life. Negative emotional experiences can produce sensations of pain in the absence of tissue damage; this can aggravate pain or reduce therapeutic effects. Mindfulness-based interventions have recently been shown to be effective for the treatment of chronic pain, and have small to moderate effects on pain and depression. Therefore, the presence of emotional disorders can seriously hinder the treatment of chronic pain; conversely, clinical treatment can relieve chronic pain through the management and regulation of emotion. Pain and depression are often treated individually and pharmacological treatment can produce side effects; therefore, the use of holistic therapy could enhance treatment outcomes.^[15]^

### 1.2 Description of the intervention

As an ancient therapeutic modality and an important part of traditional Chinese medicine (TCM), acupuncture has become a widely recognized complementary and alternative therapy in clinical practice. Acupuncture uses very fine needles to stimulate specified acupuncture points. If it is performed properly by qualified acupuncturists, acupuncture is a nontoxic treatment with an excellent safety profile (serious adverse events are rare).^[16,17]^ Acupuncture is used to treat a range of conditions, such as migraine,^[18]^ tonsillectomy pain,^[19]^ neck pain,^[20]^ knee pain,^[21]^ sciatica,^[22]^ and depression.^[23–25]^

A meta-analysis of data from nearly 18,000 randomized participants in 25 high-quality trials indicated that acupuncture is an effective treatment option for patients with back and neck pain, osteoarthritis, chronic headache, and shoulder pain.^[23]^ One randomized controlled trial (RCT) found that acupuncture significantly reduced depression at 3 months compared with usual care.^[26]^ Acupuncture can also reduce the side effects of antidepressant treatment.^[27]^ The evidence of these studies suggests that acupuncture is also an effective add-on treatment for patients with depression.^[23–28]^ One RCT to evaluate acupuncture for depression in cancer patients showed that acupuncture significantly reduced malignant-related depression and improved patient quality of life.^[29]^

### 1.3 How the intervention might work?

According to TCM theory, pain is not only a sign of discomfort but also an integral part of a particular disease or physiological malfunction. Acupuncture affects the neurovascular network and can radically change the impedances of meridians or neurovascular bundles. Therefore, both the match and mismatch of acupuncture meridian impedances with the pain source or brain impedance can reduce pain symptoms.^[30]^ Acupuncture provides overall coordination, helping to achieve the state of relative equilibrium of body and mind. In addition, some trials have investigated that acupuncture can improve depressive disorders caused by cancer or post-stroke and can act on depression by protecting nerve cells in the hippocampus.^[31–33]^ Therefore, not only where the pain is should be considered when selecting the acupoints to treat chronic pain but also the distal acupoints from TCM syndrome differentiation, which attribute depression to liver qi stagnation.^[34]^ Currently, acupuncture is a popular treatment for patients with chronic pain and depression.^[23,25–30,35]^

### 1.4 Why it is important to conduct this review?

Depression associated with chronic pain often requires long-term treatment. Compared with pharmacological therapies, acupuncture has few side effects and can significantly reduce pain and depression. There have been more than 10 systematic reviews of acupuncture for chronic pain since 2010.^[36–46]^ Unfortunately, to the best of our knowledge, there are no systematic reviews of the use of acupuncture for chronic pain with depression. Hence, a comprehensive review of acupuncture treatment of chronic pain with depression is needed and could help patients, practitioners, and health policy-makers.

### 1.5 Objectives

This review aims to systematically evaluate the efficacy of acupuncture intervention for chronic pain with depression.

## 2 Methods and analysis

### 2.1 Study registration

The protocol for this systematic review was registered with PROSPERO 2016 (registration number: CRD42016041691). This protocol report was structured according to the Preferred Reporting Items for Systematic Reviews and Meta-Analyses Protocols (PRISMA-P) statement guidelines.^[47]^ The review will be implemented according to the PRISMA statement guidelines.^[48]^

### 2.2 Inclusion criteria for study selection

#### 2.2.1 Type of study

All RCTs of acupuncture therapy for chronic pain with depression will be included in the review. Crossover studies that compared acupuncture with either sham acupuncture or non-acupuncture interventions in patients with chronic pain and depression will also be included. Nonrandomized clinical studies, cluster randomized trials, and quasi-randomized trials will be excluded.

#### 2.2.2 Type of participant

Patients diagnosed with chronic pain and depression will be included. There will be no limits on the age, sex, and source of cases. Patients experiencing only chronic pain or only depression will be excluded.

#### 2.2.3 Type of intervention

Acupuncture is defined as needle stimulation of acupoints and includes body acupuncture, scalp acupuncture, manual acupuncture, auricular acupuncture, electroacupuncture, fire needling, and plum blossom needling. The review will exclude studies that used other stimulation methods, such as acupressure, moxibustion, laser acupuncture, pharmacoacupuncture, dry needling, or transcutaneous electrical nerve stimulation. Sham acupuncture includes sham acupuncture at selected acupoints, sham acupuncture at non-acupoints, needling at inactive acupoints, nonpenetrating sham acupuncture, and pseudo-acupuncture interventions.^[49]^

We will also include trials that compared acupuncture and another typical treatment with other typical treatments alone. Control interventions will include sham/placebo acupuncture, no treatment, waiting list membership, and conventional therapies (e.g., usual care, analgesics, manual therapy).

#### 2.2.4 Type of outcome measure

##### 2.2.4.1 Primary outcomes

The primary outcome will be measured using a visual analog scale and the Hamilton Depression Scale (HAMD).

##### 2.2.4.2 Secondary outcomes

Secondary outcomes will be measured using a NRS, a verbal rating scale, and the Hospital Anxiety and Depression Scale (HADS).

### 2.3 Search methods for identification of studies

#### 2.3.1 Electronic searches

Following the core, standard, ideal search (COSI) model,^[50]^ the following electronic databases will be searched from inception to July 1, 2017: PubMed, MEDLINE, EMBASE, the Cochrane Central Register of Controlled Trials, the Allied and Complementary Medicine Database, and the Cumulative Index to Nursing and Allied Health Literature.^[51]^ We will also search the following Chinese medical databases: the China National Knowledge Infrastructure Database, the Chongqing VIP Chinese Science and Technology Periodical Database, and the Wanfang Database. We will also retrieve unpublished protocols and summary results through a search of the clinical trial registry at https://clinicaltrials.gov/.

#### 2.3.2 Searching other resources

The reference lists of potentially eligible studies and relevant systematic reviews will be manually retrieved and examined to locate additional trials. Relevant conference proceedings will also be searched to identify studies. We will also search OpenGrey.eu for potential gray literature. In addition, we plan to search relevant trial protocols using the WHO International Clinical Trial Registry Platform and ClinicalTrials.gov for ongoing and recently completed studies. We will also search conference proceedings related to the topic.

#### 2.3.3 Search strategy

The following search keywords will be used: RCT (controlled clinical trial); acupuncture (e.g., “acupuncture” or “body acupuncture” or “scalp acupuncture” or “manual acupuncture” or “auricular acupuncture”, “electroacupuncture” or “fire needling” and “plum blossom acupuncture”); chronic pain (e.g., “chronic pain” or “chronic ache” or “chronic sore” and “chronic illness”); and depression (e.g., “depression” or “mild mental disorders” or “dysthymia disorders” or “depressive disorder” or “mental illness”). For the other databases, these English search terms will be accurately translated. The search strategies for PubMed are summarized in Table 1; we will modify these strategies for other databases.

**Table 1:**
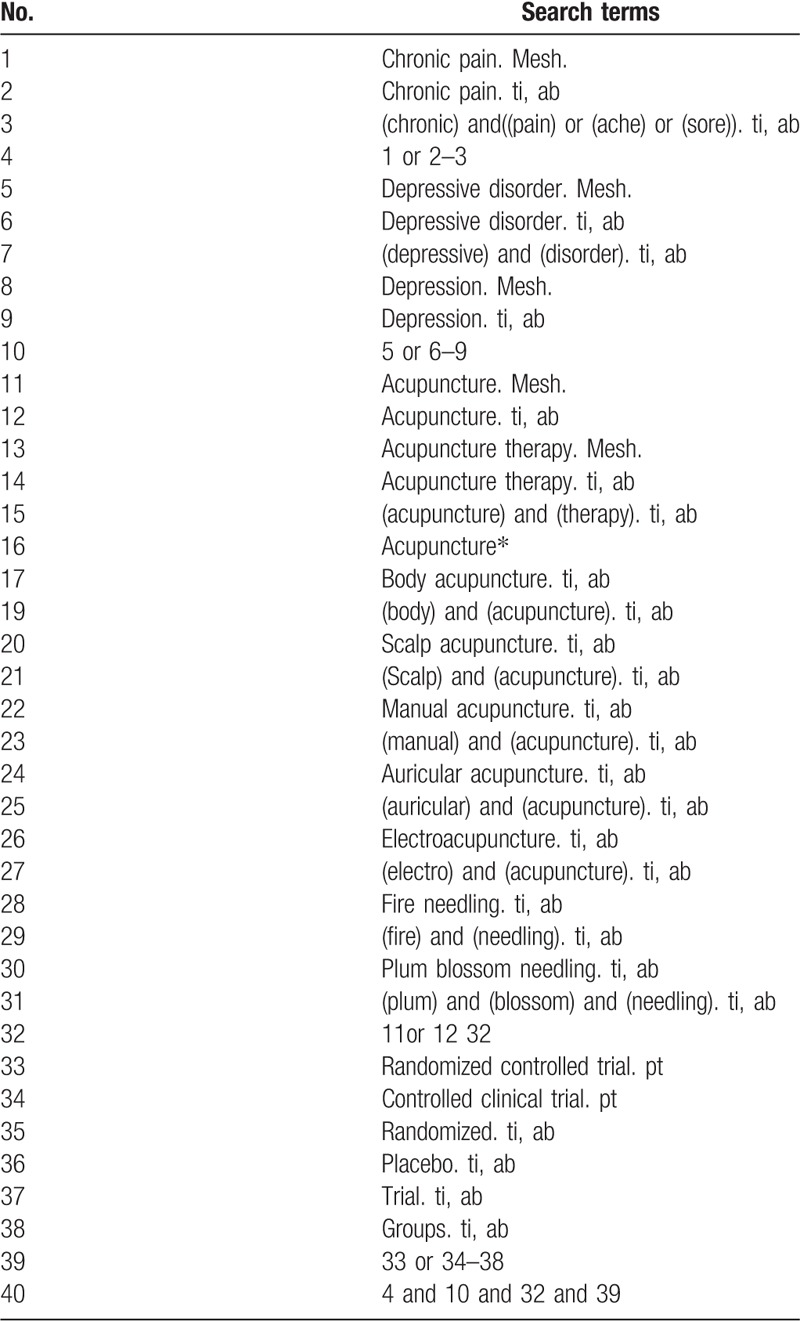
Search strategy used for PubMed database.

### 2.4 Data collection and analysis

#### 2.4.1 Selection of studies

**Figure 1:**
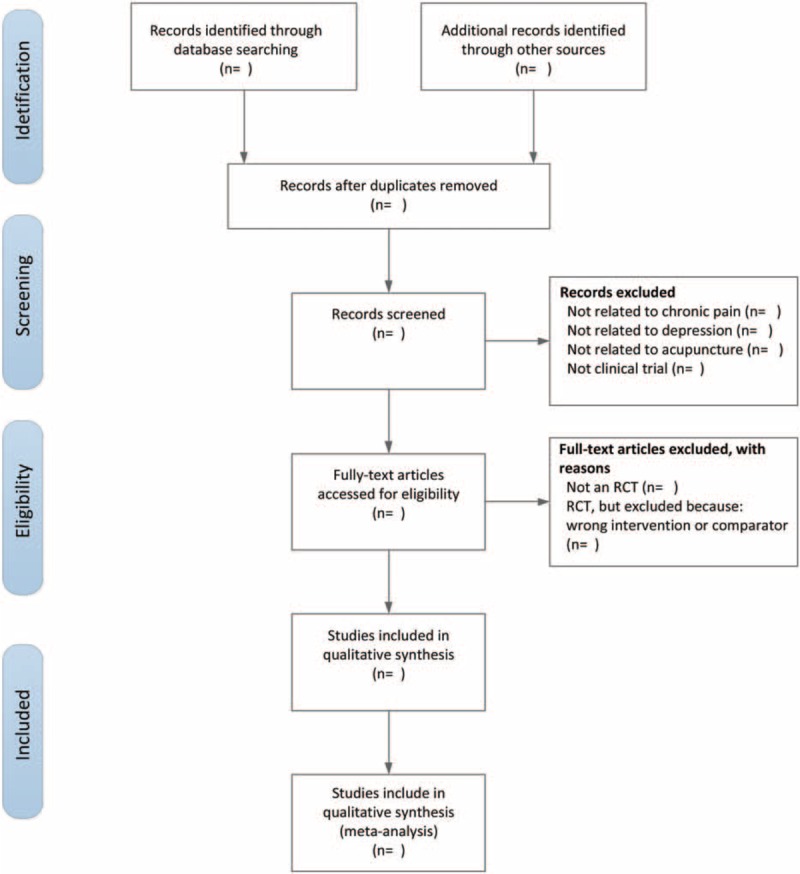
The PRISMA flow chart of the selection process.

Two reviewers will independently screen the titles and abstracts of all searched studies and eliminate duplicated or irrelevant papers. The full text of the eligible studies will be read. When the 2 reviewers cannot agree on the selection process through consultations, the third reviewer will ultimately make the decision. The primary selection process is shown in a PRISMA flow chart (Fig. 1).

#### 2.4.2 Data extraction and management

The following data will be extracted from all eligible studies by 2 independent reviewers and entered into a data extraction sheet: reference ID, first author, publication year, country, participant characteristics (e.g., average age, gender), type of intervention, type of control intervention, sample size of each intervention group, randomization, allocation concealment and blinding methods, outcome measures, main outcomes and adverse effects, duration of follow-up, type and source of financial support, and the Standards for Reporting Interventions in Controlled Trials of Acupuncture (STRICTA) checklist. In cases of insufficient data, we will contact the authors for more information. When a consensus on the data extraction cannot be obtained through consultations, the third author will make a decision.

#### 2.4.3 Assessment of risk of bias and reporting of study quality

Two review authors will independently evaluate the risk of bias using the Cochrane Collaboration's risk-of-bias assessment method and complete the STRICTA checklist for the included studies.^[52]^ The following domains will be accessed for risk of bias: sequence generation, allocation concealment, blinding of participants and personnel, blinding of outcome assessors, incomplete outcome data, selective reporting, and other issues. Trials will be assessed and categorized according to 3 levels: unclear risk, low risk, and high risk. Any discrepancies will be resolved by discussion and consensus with the third author. When a consensus on risk assessment cannot be reached by discussion, the third author will make the decision.

#### 2.4.4 Measures of treatment effect

Mean differences (MDs) with 95% confidence intervals (95% CIs) will be used to analyze continuous data. Other forms of data will be changed into MD values. Standardized MDs with 95% CIs will be used if different scales were used to measure a particular outcome variable. Dichotomous data will be analyzed using the risk ratio with 95% CIs. If significant heterogeneity is detected, a random-effects model will be used.

#### 2.4.5 Unit of analysis issues

We will focus on patients in randomized studies. If more than one acupuncture arm is used, we will conduct separate multiple meta-analyses for each treatment arm. If multiple non-acupuncture control groups are included, we will combine all control group outcomes and carry out pooled analyses of the control groups against the intervention group.

#### 2.4.6 Management of missing data

If there are missing data for the primary results, we will contact the corresponding authors to request the missing data. If the missing data cannot be obtained, the analysis will rely on the available data.

#### 2.4.7 Assessment of heterogeneity

If possible, we will use fixed-effects or random-effects meta-analyses. According to the Cochrane Handbook for Systematic Reviews of Interventions, heterogeneity can be assessed in the following ways: a visual check of the forest plot, a heterogeneity χ^2^ test, and Higgins’ *I*^2^ statistic.^[53,54]^ We will use Review Manager software (RevManV.5.3.5 for Windows; the Nordic Cochrane Centre, Copenhagen, Denmark) to obtain forest plots and heterogeneity test results. A fixed-effects model will be used to pool the data if the *P* value is larger than .10 and the *I*^2^ value is less than 50%. Otherwise, a random-effects model will be used. If the heterogeneity remains significant, subgroup analyses will be conducted. If a quantitative summary of the data still cannot be obtained, a narrative summary will be used to discuss the findings.

#### 2.4.8 Assessment of reporting biases

If more than 10 trials are included, funnel plots will be used to assess reporting biases. If funnel plot asymmetry is detected, we will try to analyze the reasons for this.

#### 2.4.9 Data synthesis

We will use RevMan for all statistical analyses. We will use either a fixed-effects model or a random-effects model, depending on the heterogeneity levels of the included studies. If considerable heterogeneity is observed, a random-effects model with 95% CIs will be used to analyze pooled effect estimates. If meaningful heterogeneity is identified that cannot be explained by any additional assessment, such as subgroup analysis, we will not attempt to perform a meta-analysis. If necessary, subgroup analysis will be performed with careful consideration of each subgroup.

#### 2.4.10 Subgroup analysis

A subgroup analysis will be conducted based on the type of control intervention and different outcomes.

#### 2.4.11 Sensitivity analysis

A sensitivity analysis will be performed according to the following criteria: sample size, heterogeneity qualities, and statistical model (random-effects or fixed-effects model).

## 3 Discussion

This study will be the first systematic review of the effectiveness of acupuncture for chronic pain with depression. The review will be divided into 4 sections: identification, study inclusion, data extraction, and data synthesis. The resulting evidence will provide important information that could benefit patients, practitioners, health policy-makers, and acupuncture practitioners.

## Acknowledgment

We thank Diane Williams, PhD, from Liwen Bianji, Edanz Group China (www.liwenbianji.cn/ac), for editing the English text of a draft of this manuscript.
